# Ultrasound grayscale image quality comparison between a 2D intracavitary transducer and a 3D intracavitary transducer used in 2D mode: A phantom study

**DOI:** 10.1002/acm2.12590

**Published:** 2019-04-19

**Authors:** Wei Zhou, Zaiyang Long, Donald J. Tradup, Scott F. Stekel, Jacinta E. Browne, Douglas L. Brown, Nicholas J. Hangiandreou

**Affiliations:** ^1^ Department of Radiology Mayo Clinic Rochester MN USA

**Keywords:** 3D transducer, image quality, objective assessment, ultrasound

## Abstract

**Purpose:**

It is unclear if a 3D transducer with the special design of mechanical swing or 2D array could provide acceptable 2D grayscale image quality for the general diagnosis purpose. The aim of this study is to compare the 2D image quality of a 3D intracavitary transducer with a conventional 2D intracavitary transducer using clinically relevant phantom experiments.

**Methods:**

All measurements were performed on a GE Logiq E9 scanner with both a 2D (IC5‐9‐D) and a 3D (RIC5‐9‐D) transducer used in 2D mode. Selection of phantom targets and acquisition parameters were determined from analysis of 33 clinical pelvic exams. Depth of penetration (DOP), contrast response, contrast of anechoic cylinders (diameter: 6.7 mm) at 1.5 and 4.5 cm depths in transverse planes, and in‐plane resolution represented by full‐width half‐maximum of pin targets at multiple depths were measured with transmit frequencies of 7 and 8 MHz. Spherical signal‐noise‐ratio (SNR) (diameter: 4 and 2 mm) at multiple depths were measured at 8 MHz.

**Results:**

RIC5‐9‐D demonstrated <8% decrease in DOP for both transmit frequencies (7 MHz: 69.7 ± 8.2 mm; 8 MHz: 64.3 ± 7.8 mm) compared with those from IC5‐9‐D (7 MHz: 73.9 ± 4.4 mm; 8 MHz: 69.4 ± 7.8 mm). A decreased anechoic contrast was observed with a 4.5 cm depth for RIC5‐9‐D (7 MHz: 23.2 ± 1.8 dB, *P* > 0.05; 8 MHz: 17.7 ± 0.9 dB, *P* < 0.01) compared with IC5‐9‐D (7 MHz: 25.9 ± 1.2 dB; 8 MHz: 21.5 ± 0.8 dB). The contrast response and spatial resolution performance were comparable between the two transducers. RIC5‐9‐D showed comparable SNR of anechoic spheres compared to IC5‐9‐D.

**Conclusions:**

2D images from a 3D probe exhibited comparable overall image quality for routine clinical pelvic imaging.

## 1 INTRODUCTION

Transvaginal ultrasound has been widely used as a routine diagnostic tool in pelvic imaging for decades.^1^, ^2^ Conventional two‐dimensional (2D) ultrasound transducers are commonly used in clinical practice for gynecology exams. Previous studies have demonstrated that volumetric three‐dimensional (3D) images could provide unique benefits of diagnosing abnormalities such as unicornuate uterus.^3^, ^4^ However, the freehand sweeping of a traditional 2D transducer to form 3D images is heavily dependent on operator's skill and is susceptible to measurement errors as the actual transducer position during the sweeping is not known by the scanner.

Alternatively, the 3D transducer techniques have been developed using either a motorized mechanism or a 2D array approach.^2^ The programmed volumetric acquisition by the 3D transducer is relatively independent of operator's skill and enables an extensive visualization of pelvic structures.^5^, ^6^ The clinical practice in our Radiology department uses 2D intracavitary transducers for routine diagnosis. A 3D acquisition may be requested by the referring physicians or radiologists based on findings from 2D images. In this case, we must either switch intracavitary transducers during the exam or schedule a second exam specifically with a 3D transducer. Both alternatives can inevitably lower the clinical efficiency and pose inconvenience for patients. It has been proposed to use 3D transducers for all transvaginal ultrasound imaging in our practice. As such, during a single visit without switching transducers, standard 2D images would be acquired with the 3D transducer. In addition, a 3D acquisition would be obtained and reconstructed using the same 3D transducer for further evaluation for each patient. Previous studies comparing 2D and 3D transducers were focused on the specific diagnostic tasks, such as the accuracy of uterus volume measurements^7^ and the visualization of intrauterine devices and deep infiltrating endometriosis.^8^, ^9^ However, it is still unclear if a 3D transducer with the special design of mechanical swing or 2D array could provide acceptable 2D grayscale image quality for the general diagnosis purpose. To accomplish comprehensive image quality assessments, it is desirable to incorporate the scanning variations in gynecology exams. The purpose of this study is to compare the 2D basic image quality of a 3D intracavitary transducer with a conventional 2D intracavitary transducer using a clinically relevant phantom study.

## 2 METHODS

### 2.1 Clinical exam survey

To ensure the clinical relevance of acquisition parameters and phantom targets used for performance measurement, 33 clinical exams using the 2D transducer model on the GE Logiq E9 ultrasound system (GE Healthcare, Milwaukee, WI, USA) for pelvic imaging from our clinical practice were randomly identified using a customized informatics toolbox.^10^ A total of 188 images from these exams were reviewed by a sonographer with more than 20 years of experience. Acquisition parameters and the characteristics of cysts (including echogenicity, size and depth), as one of the most common pathological targets,^11^ were used to determine the characteristics of targets to be measured and corresponding acquisition parameters (Table 1), including the exclusive use of harmonic imaging mode in our tests. Discussion with the radiologists also indicated that it is necessary to mimic and investigate the clinical task of searching for and counting follicles. The image depth and signal appearance information from review also indicated that the section of the phantom with 0.5 dB/cm/MHz attenuation coefficient was most appropriate for our measurements. Two acquisition modes, survey and detail characterization, were identified. The survey acquisitions were applied to search for possible occult findings or pathologies in a global manner while detail characterizations were applied for the optimal appearance of specific targets upon detection with adjusted parameters, for example, focus zone depth and gain.

**Table 1:** Review of 188 clinical ultrasound harmonic images from 33 clinical intracavitary exams

Ultrasound acquisition parameters	Transmit frequency	7 MHz (90/188)
		8 MHz (89/188)
		Others (9/188)
	Image depth	3.0–5.0 cm (102/188)
		6.0–8.0 cm (84/188)
		>8.0 cm (2/188)
Characteristics of anechoic cyst	Target depth	1.5–2.5 cm (12/19)
		3.0–5.0 cm (7/19)
	Size	Minimum: 0.8 cm
		Median: 1.9 cm
		Maximum: 9.9 cm

The distributions of image acquisition parameters and the characteristics of cysts were listed.

### 2.2 Phantom studies

All phantom image acquisitions were made with a GE Logiq E9 ultrasound system, the same system used for clinical exams in our practice. A CIRS model 040GSE phantom (CIRS Inc., Norfolk, VA, USA) was used in this study with its 0.5 dB/cm/MHz background region to perform the routine objective comparison, including depth of penetration (DOP), contrast response, contrast of anechoic cylinders, and spatial resolution, between a convex 2D (IC5‐9‐D) transducer and convex volume 3D (RIC5‐9‐D, commercially known as a 4D convex volume intracavitary transducer) transducer with motorized mechanism. Both transducers have a 145‐degree scan field of view. Transmission gel was used to couple curved probe surface with the flat surface of the phantom. All images were verified to be free from any pixel value saturation. For anechoic contrast of cylinders and spatial resolution measured at multiple depths, to mimic the clinical scenario, acquisitions were made for both survey and detail characterization modes. The acquisition parameters between the two acquisition modes for objects at different depths are identical except for focal zone and gain settings. With survey mode, an initial and standard setting of focal zone and gain was applied; while with the detail characterization mode, focal zone and gain were adjustable to achieve the optimal definition of the target at a specific depth. Table 2 illustrates the acquisition parameters for survey mode used in this study. All other acquisition parameters such as frame averaging were equivalent between the two transducers. Five repeated measurements with the CIRS phantom were acquired for each of the following tasks:

**Table 2:** Acquisition parameters for 2D image performance comparisons between the 2D (IC5‐9‐D) and 3D (RIC5‐9‐D)

Performance	Scanner control	IC5‐9‐D	RIC5‐9‐D
Depth of penetration	Transmit frequency (Harmonic)	7/8 MHz	7/8 MHz
	Dynamic range	72 dB	69 dB
	Gain	37/37	24/24
Contrast response	Transmit frequency (Harmonic)	7/8 MHz	7/8 MHz
	Dynamic range	72 dB	69 dB
	Gain	30/35	22/14
Anechoic contrast	Transmit frequency (Harmonic)	7/8 MHz	7/8 MHz
	Dynamic range	72 dB	69 dB
	Gain*	32/35	22/22
	Image depth*	5.0 cm	5.0 cm
	Focal zone*	2.5 cm	2.5 cm
Spatial resolution	Transmit frequency (Harmonic)	7/8 MHz	7/8 MHz
	Dynamic range	72 dB	69 dB
	Gain*	22/22	14/14
	Image depth*	8.0 cm	8.0 cm
	Focal zone*	4.0 cm	4.0 cm
Spherical lesion SNR	Transmit frequency (Harmonic)	8 MHz	8 MHz
	Dynamic range	72 dB	69 dB
	Gain	23	20
	Image depth*	6.0 cm	6.0 cm
	Focal zone*	3.0 cm	3.0 cm

The listed parameters were applied for the survey mode acquisitions. Gain values were adjusted to avoid any pixel value saturation for the corresponding transmit frequency which were separated by a slash (/). Controls of acquisition parameters with an asterisk (*) were optimized for each specific target when assessing performance in detailed characterization mode.

*DOP*: DOP was calculated from a pair of images (one with background base material of the phantom, the other in air) with the same acquisition settings.^12^

*Gray Contrast Response:* Contrast response was measured with the survey mode as the gray level value per dB, using the cylindrical targets with different echogenicities (−9, −6, −3, +3, and +6 dB).^13^ The echogenicity of the background base material (0 dB) was also included in the calculation.

*Anechoic Contrast:* Anechoic cylinders with 6.0 mm diameter at 1.5 and 4.5 cm depth were measured in the transverse plane for both survey and detail characterization acquisitions.^14^ The gray level difference between the anechoic cylinder and background regions were measured and converted to dB scale using the averaged gray contrast response values (measured above, in gray level per dB) for each probe and transmit frequency.

*Spatial resolution:* The profiles of high‐contrast fibers imaged in the transverse plane were measured from 1.0 to 7.0 cm depth for the survey mode, and at depths of 2.0, 4.0, and 6.0 cm for the characterization mode. The in‐plane spatial resolution was calculated as the geometric mean of the full‐width half‐maximum (FWHM) values in the axial and lateral directions.^13^

Lesion signal‐noise‐ratio (SNR) of anechoic spheres with 4.0 and 2.0 mm diameter (Gammex Sono408 phantom with 0.5 dB/cm/MHz background; Sun Nuclear Inc., Middleton, WI, USA) were measured to mimic searching follicles using both IC5‐9‐D and RIC5‐9‐D at three depths (4.0 mm: 1.5 cm, 3.0 cm, and 5.0 cm; 2.0 mm: 1.5 cm, 2.5 cm, and 3.5 cm) in survey mode. At each depth, 5 different spheres were measured with 2 orthogonal planes which yielded in total 10 measurements. 10 separate images were captured including only background materials with the same image acquisition parameters as those of the sphere images. Lesion and background images were used to calculate lesion SNR as follows:^15^, ^16^

$${\text{SNR}}_{\mathrm{Lesion}}=\frac{|{\overset{¯}{S}}_{L}-\overset{¯}{S_{B}}|}{\sqrt{\frac{\left(\sigma_{L}^{2}+\sigma_{B}^{2}\right)}{2}}}$$

$${\overset{¯}{S}}_{L}=\frac{1}{n}\sum_{i}^{n}S_{L,i}\;\;\;\;{\overset{¯}{S}}_{B}=\frac{1}{n}\sum_{1}^{n}S_{B,i}$$

where n denotes the number of targets, S_*L*,*i*_ is the average pixel value of the lesion region for *i*th target, and S_*B*,*i*_ is the average pixel value of the background region *i* defined by the same shape as for target *i*. σ_*L*_ and σ_*B*_ are calculated as the standard deviation of n values of S_*L*,*i*_ and S_*B*,*i*_, respectively.

### 2.3 DATA ANALYSIS

A commercial software package (UltraIQ, Cablon Medical B.V. Leusden, Netherlands) was used to analyze images for DOP, contrast response, and spatial resolution.^17^ Contrast of anechoic cylinders and lesion SNR were analyzed by a customized MATLAB program (MATLAB 2015a, MathWorks, Natick, MA, USA). For all image analysis, the grayscale map was analyzed and linearized to achieve a pure logarithmic conversion from the echo level to the gray level.^13^ A paired *T*‐test was conducted to compare the measurement differences between the two transducers. Statistical significance was considered with *P* < 0.05.

## 3 RESULTS

**Figure 1:**
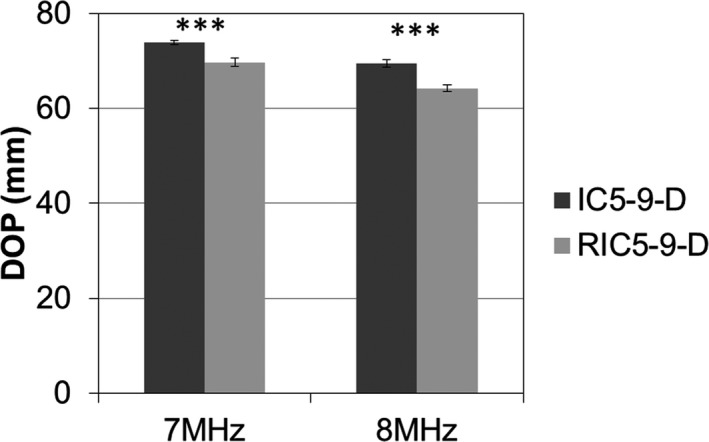
Measurements of depth of penetration (DOP) for the 2D (IC5‐9‐D) and 3D (RIC5‐9‐D) intracavitary transducers. Mean and standard deviation were illustrated for both transmit frequencies of 7 and 8 MHz. Mean ± SD with ***indicating statistical significances *P* < 0.001.

**Figure 2:**
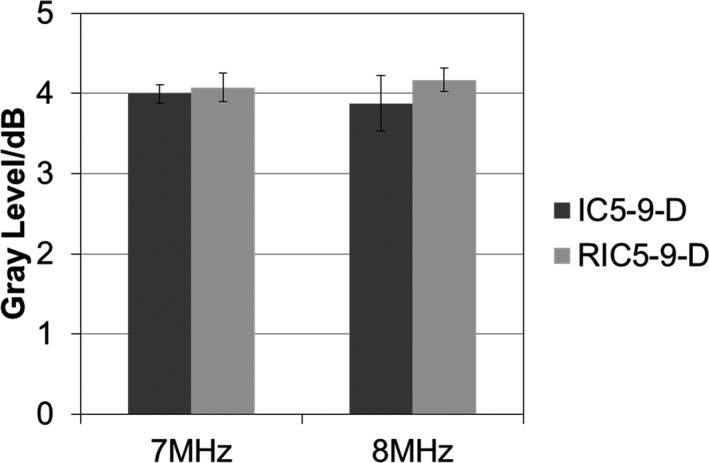
Comparison of contrast response in gray level per dB for the 2D (IC5‐9‐D) and 3D (RIC5‐9‐D) transducers. The performance of two transducers was measured by mean and standard deviation of the contrast response for transmit frequencies of 7 and 8 MHz.

The 2D images from the RIC5‐9‐D demonstrated a significantly smaller (*P* < 0.001) DOP for both transmit frequencies (7 MHz: 69.7 ± 0.8 mm; 8 MHz: 64.3 ± 0.8 mm) compared with those from the IC5‐9‐D (7 MHz: 73.9 ± 0.4 mm; 8 MHz: 69.4 ± 0.8 mm), as illustrated in Fig. 1. For gray contrast response (Fig. 2), the performance of the RIC5‐9‐D (7 MHz: 4.0 ± 0.2 gray level/dB; 8 MHz: 4.2 ± 0.1 gray level/dB) was comparable (*P* > 0.05) to that of IC5‐9‐D (7 MHz: 4.0 ± 0.1 gray level/dB; 8 MHz: 3.9 ± 0.3 gray level/dB).

**Figure 3:**
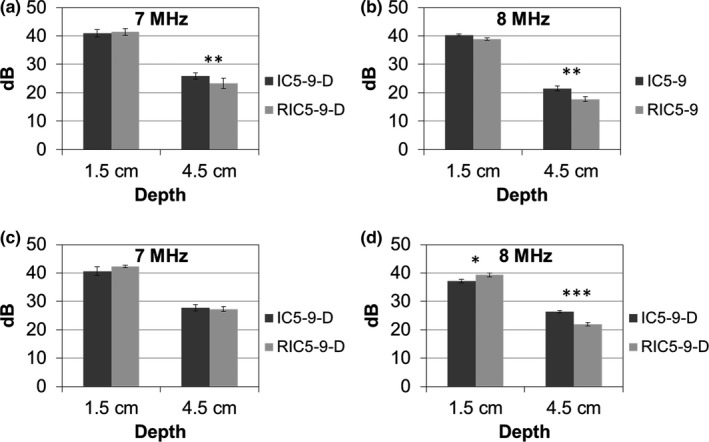
Anechoic cylinder (6.0 mm diameter) contrast in dB scale (mean and standard deviation) for the 2D (IC5‐9‐D) and 3D (RIC5‐9‐D) transducers, for the survey mode (a,b) and detail characterization acquisitions (c,d) in transverse planes. Mean ± SD with *, ** and *** indicating statistical significances *P* < 0.05, *P* < 0.01 and *P* < 0.001, respectively.

Results of anechoic cylinder contrast for the two probes and both survey and detail characterization acquisition modes are shown in Fig.**** 3. For survey mode with a 1.5‐cm‐depth target, anechoic contrast of the RIC5‐9‐D (41.4 ± 1.2 dB) was comparable (*P* > 0.05) to that of the IC5‐9‐D (7 MHz: 41.0 ± 1.3 dB) at 7 MHz; while the RIC5‐9‐D (38.9 ± 0.5 dB) showed significantly (*P* < 0.01) lower anechoic contrast than the IC5‐9‐D (40.3 ± 0.3 dB) at 8 MHz. Similar trend of anechoic contrast was observed for the 4.5‐cm‐depth cylinders for the RIC5‐9‐D (7 MHz: 23.2 ± 1.8 dB, *P* > 0.05; 8 MHz: 17.7 ± 0.9 dB, *P* < 0.01) compared with the IC5‐9‐D (7 MHz: 25.9 ± 1.2 dB; 8 MHz: 21.5 ± 0.8 dB). For the detailed characterization mode focusing on shallow (1.5 cm depth) targets, the RIC5‐9‐D demonstrated a slightly higher anechoic contrast (7 MHz: 42.3 ± 0.4 dB, *P* > 0.05; 8 MHz: 39.3 ± 0.7 dB, *P* < 0.05) compared with the IC5‐9‐D (7 MHz: 40.6 ± 1.6 dB; 8 MHz: 37.4 ± 0.7 dB) at both transmit frequencies. For deep (4.5 cm depth) targets, the RIC5‐9‐D had a significantly (*P* < 0.001) lower anechoic contrast (21.9 ± 0.6 dB) than the IC5‐9‐D (26.4 ± 0.4 dB) at 8 MHz while the performance of both transducers were similar at 7 MHz (RIC5‐9‐D: 27.3 ± 0.9 dB; IC5‐9‐D: 27.8 ± 1.1 dB; *P* > 0.05).

**Figure 4:**
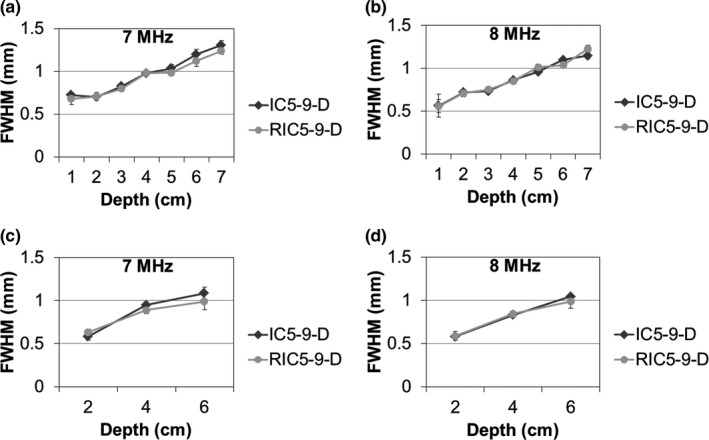
In‐plane spatial resolution for the 2D (IC5‐9‐D) and 3D (RIC5‐9‐D) transducers, for the survey mode (a,b) and detail characterization acquisitions (c,d). The in‐plane spatial resolution (mean and standard deviation) is represented by geometric mean of full‐width half‐maximum (FWHM) values at axial and lateral direction.

Figure 4 illustrated the comparison of in‐plane spatial resolution between the RIC5‐9‐D and the IC5‐9‐D. As expected, the spatial resolution for both transducer models decreased with the increased depth. For both survey and detail characterization modes, the RIC5‐9‐D showed comparable (*P* < 0.05) spatial resolution performance to the IC5‐9‐D transducer across different depths.

**Figure 5:**
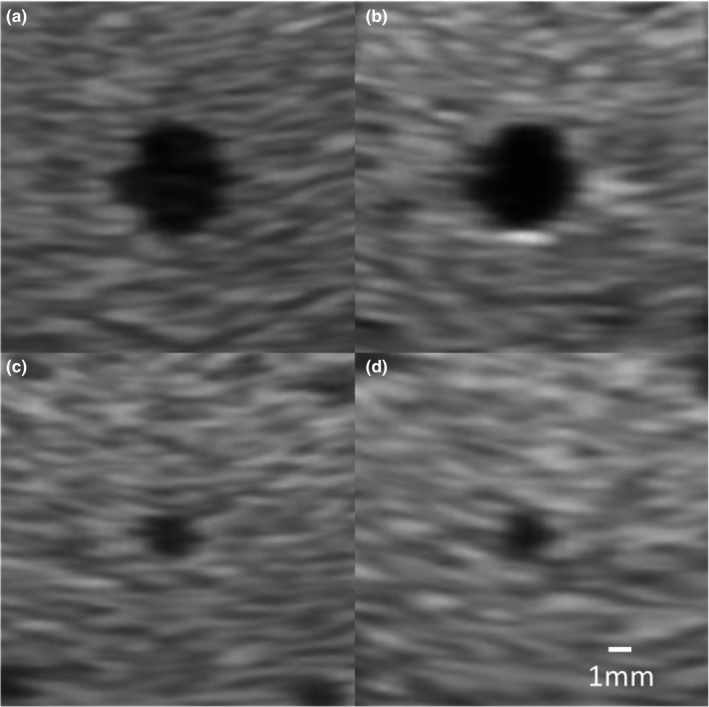
Representative images of 4‐mm anechoic spheres at 3.0 cm depth from the 2D (IC5‐9‐D) (a) and 3D (RIC5‐9‐D) (b) transducers, and 2‐mm anechoic spheres at 2.5 cm depth from the IC5‐9‐D (c) and RIC5‐9‐D (d).

**Figure 6:**
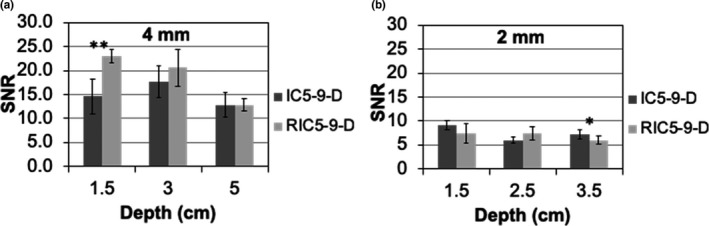
Measurements of lesion signal‐noise‐ratio (SNR) for the 2D (IC5‐9‐D) and 3D (RIC5‐9‐D) transducers from anechoic spheres with 4.0 mm (a) and 2.0 mm (b) diameter. Mean ± SD with * and ** indicating statistical significances *P* < 0.05 and *P* < 0.01, respectively.

For anechoic spheres acquired in the survey mode, representative images of 4‐mm and 2‐mm spheres from both transducers were illustrated in Fig. 5. The RIC5‐9‐D (Fig. 6) demonstrated a trend of slightly higher SNR (23.0 ± 1.4 at 1.5 cm, *P* < 0.01; 20.6 ± 3.9 at 3.0 cm, *P* > 0.05; 12.8 ± 1.3 at 5.0 cm, *P* > 0.05) for 4‐mm spherical lesions than the IC5‐9‐D (14.6 ± 3.7 at 1.5 cm, 17.7 ± 3.4 at 3.0 cm and 12.8 ± 2.6 at 5.0 cm depth). For 2‐mm spherical lesions at shallow to moderate depths, the SNR performance is, in general, comparable (*P* > 0.05) between the two transducers (RIC5‐9‐D: 7.4 ± 2.0 at 1.5 cm, 7.4 ± 1.4 at 2.5 cm; IC5‐9‐D: 8.9 at 1.5 cm, 8.5 at 2.5 cm and 6.1 at 3.5 cm depth) while at deep depths (3.0 cm), the SNR performance of IC5‐9‐D (7.2 ± 1.0) is slightly better (*P* < 0.05) than RIC5‐9‐D (6.0 ± 0.8).

## 4 DISCUSSION

It is important to assess and compare basic image quality before exclusively using the 3D transducer in clinical practice.^18^ Objective measurements have been used for ultrasound quality control programs.^13^, ^19^ Other scenarios such as acceptance tests and equipment purchase evaluations could also benefit from the objective assessment with appropriate tissue‐mimicking phantoms. Objective measurements could serve as an accurate, repeatable, and computer‐based approach for many image quality assessment cases. In this study, the objective assessments with ultrasound phantoms were used to compare the 2D image performances of a 3D (RIC5‐9‐D) and 2D (IC5‐9‐D) intracavitary transducer. An ultrasound system enables thousands of parameter combinations for acquisition following the same preset, which degraded the ability of objective assessments to predict clinical perception.^20^, ^21^ A careful review of clinical exams was conducted first to ensure that phantom scanning was performed in a manner very similar to the clinical scanning, and that the phantom targets used for our performance measurements correlated with actual clinical imaging tasks. This made the performance measurements as relevant to actual clinical practice as possible.

Our data showed, in general, that the RIC5‐9‐D provided comparable imaging performance to the IC5‐9‐D. The approximately 6–7% or <5 mm decrease in DOP for RIC5‐9‐D compared to IC5‐9‐D could be due to the oil in typical 3D transducer designs which couples the array with the scanning window.^22^ As our clinical exam survey results showed, the majority of clinical interest regions or targets for pelvic intracavitary exams are superficial (<5.0 cm depth). At both transmit frequencies, the RIC5‐9‐D could still provide sufficient signals with meaningful echo information for clinical intracavitary exams. Therefore, the slight decrease in DOP would not pose a substantial impediment for using the RIC5‐9‐D.

In this study, we found the RIC5‐9‐D had a slightly worse contrast of anechoic cylinders and similar SNR of anechoic spheres, compared to the IC5‐9‐D. Several reasons could explain the observed discrepancies between contrast and SNR results. First, contrast measurements do not take target size into calculation, while SNR does. Second, the SNR of spherical targets were comprehensively affected by the anechoic contrast as well as the spatial resolution performances.^23^ In addition, all the SNR results were above the visual detection threshold that the small differences in contrast might not be clinically important.^24^

This study established the framework of utilizing physics tests for evaluating ultrasound system performance. There are a few limitations of this study that should be noted. First, only one of each transducer model was investigated and both transducers used are relatively new and visually intact. Future study could consider including more transducers with a variety of usage time. Second, this study only included anechoic targets to mimic cystic lesion while targets with other echogenicity should be considered if clinically relevant in certain practice. Third, although physics testing can benchmark the fundamental technical probe performance; direct comparisons of clinical images obtained with both probe models in the same patients should also be reviewed. This step is currently underway in our practice. The final decision on which probe to use in routine practice will also be dependent on several other factors, including the cost of replacing all 2D transducers and adding 3D imaging capability upgrades to more scanners. Finally, the image quality and measurement accuracy using the 3D mode should be evaluated as the next step.

## 5 CONCLUSION

The 2D grayscale image quality of a conventional 2D intracavitary and 3D intracavitary transducer was compared in this study. We demonstrated that the 3D RIC5‐9‐D was comparable to IC5‐9‐D in terms of 2D image quality. With further confirmation from patient image comparison, RIC5‐9‐D intracavitary transducers could routinely be used in all pelvic exams in the practice.

## CONFLICT OF INTERESTS

No conflict of interests.
